# Supplementary material for: A Risk Factor Analysis of SARS-CoV-2 Infection in Animals in COVID-19-Affected Households
Source: Viruses. 2023 Mar 11;15(3):731. doi: 10.3390/v15030731 (PMC10051903; doi:10.3390/v15030731)
Supplement: Supplementary file 1 [file viruses-15-00731-s001.zip › viruses-2239989-supplementary.pdf]

Supplements

Questionnaire for SARS-CoV-2 positive pet owners

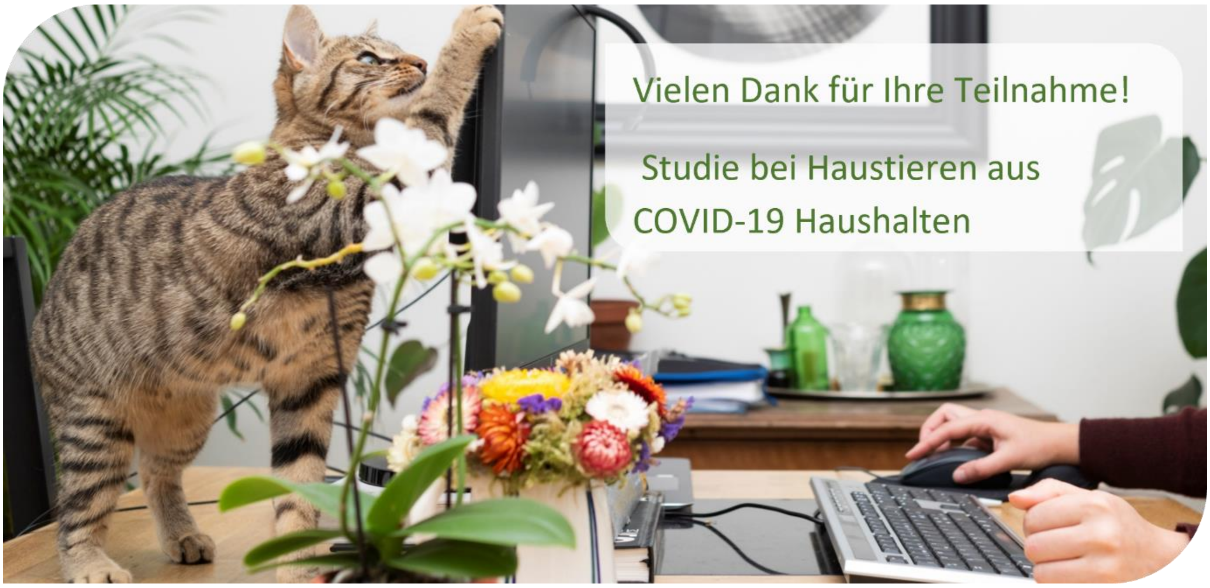

The study "Investigation on SARS-CoV-2 in pets" collects data on pet handling and hygiene in COVID-19 households. The questionnaire is aimed at SARS-CoV-2 positive pet owners or pet owners who have tested positive for SARS-CoV-2 within the last 6 months.

The questionnaire will be completed by one of the persons in the household who has COVID-19 ("participant"). This person will also be asked to fill in the details of your household members (housemate 1, 2, 3, 4). PLEASE ASSIGN THE SAME NUMBER TO YOUR HOUSEMATES ACCORDING TO THE CONSENT FORM!

The questionnaire has been prepared for a household consisting of a maximum of 5 persons. If there are more than 5 people living in the household, please contact us.

The data will be kept strictly confidential and anonymous.

Thank you very much!

A. General questions for household members

1a. Please enter your study number, which you will find on the consent form.
USZ-_______________

2a. Please enter today's date. (Date format: dd.mm.yyyy)
____

3a. How many pets live in your household? (One choice per row)

	0	1	2	3	4	5	6	7
Cats								

Dogs								
Other animals								

4a. How many other household members live in your household besides you? Please choose one of the following answers:
0; 1; 2; 3; 4; 5 (as dropdown menu)

a. Age of the household members (one choice per row)

	Under 18 years	18 – 30 years	31 – 40 years	41 – 60 years	Over 60 years
Participant					
Roommate 1					
Roommate 2					
Roommate 3					
Roommate 4					

b. Gender of household members (one choice per row)

	male	female	diverse	No answer
Participant				
Roommate 1				
Roommate 2				
Roommate 3				
Roommate 4				

c. Which household members take care of the pet? (one choice per row)

	Yes	No	No answer
Participant			
Roommate 1			

Roommate 2			
Roommate 3			
Roommate 4			

d. Are there any household members who are at risk*? (one choice per row)

	Yes	No	No answer
Participant			
Roommate 1			
Roommate 2			
Roommate 3			
Roommate 4			

*Risk patients are e.g. persons >65 years, pregnant women, immunocompromised persons, persons with diabetes, high blood pressure, cardiovascular diseases or cancer.

e. Which household member(s) has/have tested positive for COVID-19? Please indicate the date of the first positive COVID-19 test as a comment.

	Yes	Date of first positive COVID-19 test
Participant		
Roommate 1		
Roommate 2		
Roommate 3		
Roommate 4		

5a. Did you know that there are recommendations for owners of dogs and cats in connection with COVID-19 from the Federal Food Safety and Veterinary Office (FSVO)? Please choose one of the following answers:
Yes/No

B. General hygiene

6a. Hand washing behaviour BEFORE COVID-19 diagnosis: How many times a day do you wash your hands with soap?

	Never	1-2 times	3-4 times	5-6 times	7-12 times	more than 12 times	no answer
Participant							
Roommate 1							
Roommate 2							
Roommate 3							
Roommate 4							

7a. Has your hand hygiene changed since a household member got COVID-19?

	YES, I wash/disinfect my hands more often than before.	NO, unchanged.	No answer
Participant			
Roommate 1			
Roommate 2			
Roommate 3			
Roommate 4			

8a. Behaviour towards coughing/sneezing BEFORE COVID-19 diagnosis: do you wash your hands after coughing/sneezing?

	Coughing					Sneezing				
	always	often	rarely	never	No answer	always	ofte n	rarely	never	No answer
Partici pant										
Room mate 1										
Room mate 2										
Room mate 3										
Room mate 4										

	Coughing					Sneezing				
	always	often	rarely	never	No answer	always	ofte n	rarely	never	No answer
Partici pant										
Room mate 1										
Room mate 2										
Room mate 3										
Room mate 4										

9a. Behaviour towards coughing/sneezing BEFORE COVID-19 diagnosis: Do you cough/sneeze into a tissue?

10a. Has your behaviour towards coughing/sneezing changed during the COVID-19 illness?

C. Pet hygiene

11a. How often do you wash your pet(s) toys?

	daily	weekly	Every 2 weeks	monthly	Half-yearly	Less often	My pet(s) has/have no toys
before COVID-19							
during COVID-19							

12a. How often is your pet(s) bed washed?

	daily	weekly	Every 2 weeks	monthly	Half-yearly	Less often	My pet(s) has/have no bed
before COVID-19							
during COVID-19							

13a. How often is your pet(s) food bowl washed?

	I wash/disinfect my hands more often afterwards			I cough/sneeze more often into a handkerchief		
	YES	NO, my behaviour is unchanged.	no answer	YES	NO, my behaviour is unchanged.	no answer
Participant						
Room mate 1						
Room mate 2						
Room mate 3						
Room mate 4						

	daily	weekly	Every 2 weeks	monthly	Half-yearly	Less often	My pet(s) has/have no bowl
before COVID-19							
during COVID-19							

14a. What do you use to clean your pet(s) food bowl?
Please select the answers that apply (multiple answers possible):

	with water
	with soap and water
	with a separate cloth
	with the same cloth I use for my dishes
	in the dishwasher
	with a special detergent/disinfectant
	my pet(s) does not have a food bowl

D. Interaction with the pet(s) - Types of contact between household members and the pet(s)

Please indicate how often which interaction takes place between which household member and the pet(s).

15a. Direct contact time with the pet(s) is usually:

	Over 8 hours a day	2-8 hours a day	1-2 hours a day	10 min to 1 hour a day	under 10 minutes a day	no answer
Participant						
Roommate 1						
Roommate 2						
Roommate 3						
Roommate 4						

16a. Did you change your behaviour towards the animal when a household member became ill with COVID-19?

	Yes, contact more intense than before	Yes, contact was limited	No, contact unchanged as before	I don`t know
Participant				
Roommate 1				
Roommate 2				
Roommate 3				
Roommate 4				

17a. During/since the COVID-19 diagnosis, does another person in the household take more care of the pet? Please choose one of the following answers:

	Participant
	Yes, housemate 1
	Yes, housemate 2
	Yes, housemate 3
	Yes, housemate 4
	No, the care of the pet is distributed as before
	A person not living in the household takes care of the pet
	The pet was taken to an external institution (animal shelter/boarding kennel)
	Other:

(Animal-specific part: The owner gets the species-specific section for each animal according to the indication above.)

E. Animal-specific questions for cat owners (Cat 1)

Please answer the questionnaire for one cat only, if you own more cats you will be redirected later.

18a. Indicate the assigned ID number and the name of the animal.
ID number:__________
Name:_________

19a. Please indicate the sex of your cat.
male; male neutered; female; unknown (as a dropdown menu)

20a. Please indicate the age of your cat.
1; 2; 3; 4; 5; 6; 7; 8; 9; 10 ; 11; 12; 13; 14; 15; 16; 17; 18; 19; 20, over 20; unknown

21a. Where is the main residence of your cat? Please choose one of the following answers:

	My cat stays exclusively in the flat

	My cat lives exclusively in the flat and has access to the balcony/terrace.
	My cat spends less than 2 hours per day* outside.
	My cat spends 2-6 hours per day* outside.
	My cat spends 12 hours per day* outside.
	My cat only comes to feed and otherwise stays outside.

*Interpretation of a day of 24h

22a. Does your pet have any known pre-existing conditions*? And is it under any therapy for this? If yes, please enter them in the comment field. Please choose one of the following answers:number

	No, my cat has no pre-existing conditions
	Yes, my cat has the following pre-existing conditions. Please enter your comment here:
	I do not know if my cat has any pre-existing conditions.

* e.g.: Diabetes, heart disease, respiratory disease, cat flu, cancer etc.

23a. Does your cat show one or more of the following symptoms since COVID-19 diagnosis in a household member? Please select the answers that apply:

	sneezing
	dry cough
	cough with expectoration of mucus
	rapid or labored breathing (respiratory rate over 40/min if countable)
	Breathing sounds
	Nasal discharge
	Eye discharge
	Salivation
	Vomiting
	Diarrhea
	tiredness, listlessness
	reduced appetite
	I do not know
	No, my animal has no symptoms

24a. Have you seen a veterinarian for these symptoms? If yes: What was the diagnosis and treatment?

	No, I have not seen a veterinarian.
	Yes, I have seen a veterinarian. The diagnosis and treatment were: Please enter your comment here:

25a. How often do you have very close contact with your cat ?

	very often (several times a day)	often (daily)	rarely	never	no answer
Have hands licked off					
Have face licked off					
Giving kisses					
Giving treats					

26a. How often do you have the following types of contact with your cat?

	Over 8 hours a day	2-8 hours a day	1-2 hours a day	10 min to 1 hour a day	under 10 minutes a day	never	no answer
sleeping in the same bed							
Lying together on the sofa							
Stroking/cuddling							
Walking							
Playing							
Staying in the same room							

27a. How often do you have indirect contact with your cat?

	Over 2x daily	1-2x daily	over 1x per week	1x per week	1x per month	rarely	never	no answer
Give food								
Clean up/remove droppings (garden/dung box)								
Cleaning the droppings box (e.g. changing sand,								

washing, disinfecting)								

F. Animal-specific questions for dog owners (Dog 2)

Please answer the questionnaire for one dog only, if you own more cats you will be redirected later.

18b. Indicate the assigned ID number and the name of the animal.
ID number:__________
Name:_________

19b. Please indicate the sex of your dog.
male; male neutered; female; unknown (as a dropdown menu)

20b. Please indicate the age of your dog.
1; 2; 3; 4; 5; 6; 7; 8; 9; 10 ; 11; 12; 13; 14; 15; 16; 17; 18; 19; 20, over 20; unknown

21b. How much time of the day does your dog spend outside?

	My dog spends less than 1h per day* outside.
	My dog spends less than 2 hours per day* outside.
	My dog spends 2 -6 hours per day* outside.
	My dog spends 12 hours per day* outside.
	My dog spends the whole day outside.

*Interpretation of a day of 24h

22b. Does your pet have any known pre-existing conditions*? And is it under any therapy for this? If yes, please enter them in the comment field. Please choose one of the following answers:

	No, my dog has no pre-existing conditions
	Yes, my dog has the following pre-existing conditions. Please enter your comment here:
	I do not know if my dog has any pre-existing conditions.

* e.g.: Diabetes, heart disease, respiratory disease, cat flu, cancer etc.

23b. Does your dog show one or more of the following symptoms since COVID-19 diagnosis in a household member? Please select the answers that apply:

	sneezing
	dry cough
	cough with expectoration of mucus

	rapid or labored breathing (respiratory rate over 40/min if countable)
	Breathing sounds
	Nasal discharge
	Eye discharge
	Salivation
	Vomiting
	Diarrhea
	tiredness, listlessness
	reduced appetite
	I do not know
	No, my animal has no symptoms

24b. Have you seen a veterinarian for these symptoms? If yes: What was the diagnosis and treatment?

	No, I have not seen a veterinarian.
	Yes, I have seen a veterinarian. The diagnosis and treatment were:<br>Please enter your comment here:

25b. How often do you have very close contact with your dog ?

	very often (several times a day)	often (daily)	rarely	never	no answer
Having hands licked off					
Having hands licked off					
Giving kisses					
Giving treats					

26b. How often do you have the following types of contact with your dog?

	Over 8 hours a day	2-8 hours a day	1-2 hours a day	10 min to 1 hour a day	under 10 minutes a day	never	no answer
sleeping in the same bed							
Lying together on the sofa							
Stroking/cuddling							
Walking							
Playing							
Staying in the same room							

27b. How often do you have indirect contact with your dog?

	Over 2x daily	1-2x daily	over 1x per week	1x per week	1x per month	rarely	never	no answer
Give food								
Clean up/remove feces								

G. Animal-specific questions owners of other pets (other pet 1)

Please answer the questionnaire for one other animal only, if you own more cats you will be redirected later.

18c. Indicate the assigned ID number and the name of the animal.

ID number:__________

Name:_________

19c. What species is your animal?

Rabbit; guinea pig; hamster; turtle; mouse; rat; bird; other (as a dropdown menu)

20c. Please indicate the sex of your animal.

male; male neutered; female; unknown (as a dropdown menu)

21c. Please indicate the age of your animal.

1; 2; 3; 4; 5; 6; 7; 8; 9; 10 ; 11; 12; 13; 14; 15; 16; 17; 18; 19; 20, over 20; unknown

22c. The animal lives with me in my flat/house?

If the animal does not live in the apartment/house with you, please state the animal`s whereabouts in the comments field.

	Yes
	No but:

23c. How much time of the day does your animals spend outside?

	My pet never stays outside.
	My pet spends less than 1h per day* outside.
	My pet spends less than 2 hours per day* outside.

	My pet spends 2-6 hours per day* outside.
	My pet spends 12 hours per day* outside.
	My pet spends the whole day outside.

*Interpretation of a day of 24h

24c. Does your pet have any known pre-existing conditions*? And is it under any therapy for this? If yes, please enter them in the comment field. Please choose one of the following answers:

	No, my pet has no pre-existing conditions
	Yes, my pet has the following pre-existing conditions. Please enter your comment here:
	I do not know if my pet has any pre-existing conditions.

* e.g.: Diabetes, heart disease, respiratory disease, cat flu, cancer etc.

25c. Does your pet show one or more of the following symptoms since COVID-19 diagnosis in a household member? Please select the answers that apply:

	sneezing
	dry cough
	cough with expectoration of mucus
	rapid or labored breathing (respiratory rate over 40/min if countable)
	Breathing sounds
	Nasal discharge
	Eye discharge
	Salivation
	Vomiting
	Diarrhea
	tiredness, listlessness
	reduced appetite
	I do not know
	No, my animal has no symptoms

18c. Have you seen a veterinarian for these symptoms? If yes: What was the diagnosis and treatment?

	No, I have not seen a veterinarian.
	Yes, I have seen a veterinarian. The diagnosis and treatment were: Please enter your comment here:

19c. How often do you have very close contact with your pet ?

	very often	often (daily)	rarely	never	no answer

	(several times a day)				
Having hands licked off					
Having face licked off					
Giving kisses					
Giving treats					

20c. How often do you have the following types of contact with your pet?

	Over 8 hours a day	2-8 hours a day	1-2 hours a day	10 min to 1 hour a day	under 10 minutes a day	never	no answer
sleeping in the same bed							
Lying together on the sofa							
Stroking/cuddling							
Walking							
Playing							
Staying in the same room							

21c. How often do you have indirect contact with your pet?

	Over 2x daily	1-2x daily	over 1x per week	1x per week	1x per month	rarely	never	no answer
Give food								
Clean up/remove droppings (clean enclosure/stable/cage etc.)								
